# Combined RBE and OER optimization in proton therapy with FLUKA based on EF5‐PET

**DOI:** 10.1002/acm2.14014

**Published:** 2023-05-10

**Authors:** Helge Henjum, Tordis Johnsen Dahle, Andrea Mairani, Sara Pilskog, Camilla Stokkevåg, Camilla Grindeland Boer, Kathrine Røe Redalen, Heikki Minn, Eirik Malinen, Kristian Smeland Ytre‐Hauge

**Affiliations:** ^1^ Department of Physics and Technology University of Bergen Bergen Norway; ^2^ Department of Oncology and Medical Physics Haukeland University Hospital Bergen Norway; ^3^ Centro Nazionale di Adroterapia Oncologica (CNAO Foundation) Pavia Italy; ^4^ Heidelberg Ion Beam Therapy Center (HIT) Heidelberg Germany; ^5^ Department of Physics Norwegian University of Science and Technology Trondheim Norway; ^6^ Department of Oncology and Radiotherapy Turku University Hospital Turku Finland; ^7^ Turku PET Centre University of Turku Turku Finland; ^8^ Department of Physics University of Oslo Oslo Norway; ^9^ Department of Medical Physics Oslo University Hospital Oslo Norway

**Keywords:** hypoxia, oxygen enhancement ratio, proton therapy, relative biological effectiveness

## Abstract

**Introduction:**

Tumor hypoxia is associated with poor treatment outcome. Hypoxic regions are more radioresistant than well‐oxygenated regions, as quantified by the oxygen enhancement ratio (OER). In optimization of proton therapy, including OER in addition to the relative biological effectiveness (RBE) could therefore be used to adapt to patient‐specific radioresistance governed by intrinsic radiosensitivity and hypoxia.

**Methods:**

A combined RBE and OER weighted dose (ROWD) calculation method was implemented in a FLUKA Monte Carlo (MC) based treatment planning tool. The method is based on the linear quadratic model, with *α* and *β* parameters as a function of the OER, and therefore a function of the linear energy transfer (LET) and partial oxygen pressure (pO_2_). Proton therapy plans for two head and neck cancer (HNC) patients were optimized with pO_2_ estimated from [^18^F]‐EF5 positron emission tomography (PET) images. For the ROWD calculations, an RBE of 1.1 (RBE_1.1,OER_) and two variable RBE models, Rørvik (ROR) and McNamara (MCN), were used, alongside a reference plan without incorporation of OER (RBE_1.1_).

**Results:**

For the HNC patients, treatment plans in line with the prescription dose and with acceptable target ROWD could be generated with the established tool. The physical dose was the main factor modulated in the ROWD. The impact of incorporating OER during optimization of HNC patients was demonstrated by the substantial difference found between ROWD and physical dose in the hypoxic tumor region. The largest physical dose differences between the ROWD optimized plans and the reference plan was 12.2 Gy.

**Conclusion:**

The FLUKA MC based tool was able to optimize proton treatment plans taking the tumor pO_2_ distribution from hypoxia PET images into account. Independent of RBE‐model, both elevated LET and physical dose were found in the hypoxic regions, which shows the potential to increase the tumor control compared to a conventional optimization approach.

## 1 INTRODUCTION

Hypoxic cells are more radioresistant than well‐oxygenated cells, and tumor hypoxia (insufficient oxygen supply) is generally associated with poor treatment outcome.^1^ The increased radioresistance due to hypoxia is commonly quantified by the oxygen enhancement ratio (OER), which is the ratio of the dose at a given oxygen pressure to that at a standard oxygen pressure producing the same biological effect. While several methods have been proposed to account for local variations in tumor OER in treatment planning,^2^, ^3^, ^4^, ^5^, ^6^, ^7^ none of these are currently available in commercial clinical treatment planning systems (TPSs).

Hypoxia is a known cause of clinical radioresistance in head and neck cancer (HNC), and there are x‐ray hypoxia dose painting trials showing how dose escalation in hypoxic volumes could improve treatment outcome.^8^ For ions heavier than protons, effective dose escalation to account for hypoxia has been performed in silico^3^, ^6^, ^7^ using kill painting, where both the dose and the LET are used to achieve the same cell killing as in normoxic tissue, as first proposed by Scifoni et al.,^6^ and further by Tinganelli et al.^7^ Both these studies used the TRiP98 software^9^, ^10^ for treatment planning and OER models based on heavy ion experiments. The kill painting methodology was further explored in a clinical treatment plan studied by Sokol et al.^11^ utilizing a synthetic pO_2_ map emulating hypoxia imaging and forming the basis for multi‐ion optimization. Antonovic et al.^12^ studied the role of hypoxia and local oxygenation changes with carbon ions in a water phantom with simulated pO_2_ values. For protons, Köthe et al.^13^ showed how contour‐based dose escalation, based on a PET‐uptake threshold, with protons in non‐small lung cancer patients could improve the tumor control probability. Other techniques include linear energy transfer (LET)‐painting,^4^ which restricts high‐LET radiation to volumes found to be hypoxic, while applying lower LET radiation to normoxic tissues.

Currently in proton therapy, a constant relative biological effectiveness (RBE) of 1.1 is applied, but several phenomenological and mechanistic RBE models have been proposed to account for the variable RBE in aerobic tumors.^14^, ^15^, ^16^ RBE and OER weighted dose (ROWD) optimization, giving hypoxic regions a physical dose and LET boost similar to hypoxia kill and LET painting, could provide an improved tumor effect. ROWD optimization has the benefit of being flexible to the selection of RBE model with more personalized radiobiological parameters. Optimization based on clinically relevant hypoxia imaging still needs exploration for proton therapy and, to our knowledge, no PET‐based voxel‐based ROWD optimization applying phenomenological RBE models for protons have as yet been presented.

In this study we perform [^18^F]‐EF5 positron emission tomography (PET) guided voxel‐by‐voxel ROWD optimization with protons in two HNC patient cases. In our previous study,^17^ we created a MC based tool which includes hypoxia and RBE in recalculations of the ROWD. This tool could estimate the ROWD using variable RBE based on the linear quadratic model with OER‐dependent radiosensitivity parameters *α* and *β*. In this study, we developed a methodology for ROWD optimization with to use different RBE‐models within the FLUKA based treatment planning tool. The work may open for novel applications employing a wider range of radiobiological parameters in treatment planning.

## 2 MATERIALS AND METHODS

### 2.1 Estimation of the oxygen enhancement ratio

The OER was estimated using a modification of the OER model by Wenzl and Wilkens,^18^ using only in vitro proton data, as described in our previous study^17^:

(1) $$\begin{array}{ccc} & & \mathrm{OER}\left(L,\;p_{h}\right)=\\ & & \;\frac{\sqrt{\alpha^{2}\left(L,p_{h}\right)-4\beta \left(p_{h}\right)\cdot \ln\left(0.1\right)}-\alpha \left(L,p_{h}\right)}{\sqrt{\alpha^{2}\left(L,p_{a}\right)-4\beta \left(p_{a}\right)\cdot \ln\left(0.1\right)}-\alpha \left(L,p_{a}\right)}\cdot \frac{\beta \left(p_{a}\right)}{\beta \left(p_{h}\right)}\; \end{array}$$

where, *L* is the dose‐averaged linear energy transfer (LET_d_), *p_h_* is the partial pressure of oxygen (pO_2_) in a given voxel in the patient or phantom and the OER is taken at 10% cell survival. α(L,p) and β(p) are radiosensitivity parameters given by

(2) $$\alpha \left(L,p\right)=\frac{\left(a_{1}+a_{2}\cdot L\right)\cdot p+\left(a_{3}+a_{4}\cdot L\right)\cdot K}{p+K},$$

(3) $$\sqrt{\beta \left(p\right)}=\frac{b_{1}\cdot p+b_{2}\cdot K}{p+K},$$

where *p* is the pO_2_ and *K* is a parameter set to 3 mmHg.^19^ The model parameters were found by non‐linear least square curve fit of in vitro proton data to be: *a*_1_=0.10 Gy^−1^, *a*_2_=0.0010 µm/(Gy·keV), *a*_3_=0.010 Gy^−1^, *a*_4_=0.0100 µm/(Gy·keV), *b*_1_=0.765 Gy^−1^, and *b*_2_=0.273 Gy^−1^.^17^ *p_a_* was set to 30 mmHg.

The pO_2_ values in the patients were estimated on a voxel‐by‐voxel basis from PET images with [^18^F]‐EF5 as hypoxia tracer, as described in Dahle et al.^15^ The PET images were acquired using a GE D690 PET/CT scanner (General Electric Medical Systems, Milwaukee, WI, USA) at Turku University Hospital, Finland. For information on the synthesis of [^18^F]‐EF5 and imaging protocols, see Silvoniemi et al.^20^

### 2.2 RBE and OER weighted dose calculations

The ROWD (*D*_OER,RBE_) was estimated as follows:

(4) $$D_{\mathrm{OER},\mathrm{RBE}}=D\times \frac{1}{D_{p}}\left(\sqrt{{\left(\frac{\alpha_{x}}{2\beta_{x}}\right)}^{2}+\frac{\alpha_{h}D_{p}+\beta_{h}D_{p}^{2}}{\beta_{x}}}-\frac{\alpha_{x}}{2\beta_{x}}\right),$$

as described in detail in our previous study.^17^ Here, $\alpha_{x}$ and $\beta_{x}$ are the aerobic photon radiosensitivity parameters, $\alpha_{h}$ and $\beta_{h}$ are the pO_2_‐dependent proton radiosensitivity parameters, *D* is defined as the total physical dose from protons and secondary particles and *D_p_* is the physical dose from protons from primary and secondary protons.

The hypoxic proton radiosensitivity parameters are functions of the OER:

(5) $$\alpha_{h}=\alpha_{RBE}/\mathrm{OER}\left(L,p_{h}\right),$$

(6) $$\beta_{h}=\beta_{RBE}/{\mathrm{OER}}^{2}\left(L,p_{h}\right),$$

where $\alpha_{RBE}$ and $\beta_{RBE}$ are the biological parameters for the variable RBE models. The aerobic radiosensitivity parameters ($\alpha_{x}$, $\beta_{x}$, $\alpha_{RBE}$ and $\beta_{RBE}$) can be defined according to most existing variable RBE models based on the linear quadratic model with parameters based on aerobic proton and photon in vitro data (see overview in Rørvik et al.^14^). Details on how the $\alpha_{RBE}$ and $\beta_{RBE}$ are calculated for the different models can be found in the appendix. Then, the hypoxic radiosensitivity parameters for protons, $\alpha_{h}$ and $\beta_{h}$, can be estimated from Equations (5) and (6). We applied the $\alpha_{x}$, $\beta_{x}$, $\alpha_{RBE}$, and $\beta_{RBE}$ parameters, as well as a constant RBE of 1.1 and the Rørvik variable RBE model (ROR), described in our previous study for estimating the ROWD for ROR and RBE_1.1_.^17^ Additionally, in this study we included a second RBE model to evaluate the consistency of the ROWD optimization results across different RBE models. The McNamara model (MCN). The MCN model is based on the largest existing cell‐line library, and is based on dose averaged LET‐values as opposed to the LET‐spectrum as in the ROR model, and the belonging data are given in the Supplementary Materials.^21^ In the RBE calculation of both models, the LET was calculated from the primary and secondary protons.

### 2.3 Implementation of the model in the FLUKA MC based treatment planning tool

We implemented the ROWD calculation described in the previous chapter with the prototype optimization algorithm described by Mairani et al.^22^, ^23^ and further modified it to fit our in‐house dose verification system, also including multiple RBE models.

The FLUKA MC based treatment planning tool is divided into two main steps; an initial FLUKA simulation and the optimization process. The initial FLUKA simulation was run applying a first approximation of the treatment plan, in our study given by RBE_1.1_ plans. In this simulation, we score the parameters needed for calculating and optimizing the ROWD, which includes the biological variables $\alpha_{h}D_{p}$, $\sqrt{\beta_{h}}D_{p}$ and the dose *D*.

The output from the initial FLUKA run, as well as the constants *α_x_* and *β_x_* and scripts describing the planning target volume (PTV) and organs at risk (OAR) of interest, was then used as input to the optimizer. In the optimizer, the ROWD was estimated on a voxel‐by‐voxel basis according to Equation (4), thus the parameters are not explicitly optimized. The optimization was then done using the dose difference optimization algorithm described in Mairani et al.,^22^ using the cost function found in the Supplementary Materials. Another algorithm, a plain gradient method, were also tested which provided similar optimization outcome.

In short, the optimizer aims to create a treatment plan with a homogeneous ROWD to the PTV as close to the prescribed dose as possible, while constraining the dose to the OARs satisfactorily. The output from the optimizer is the pencil beams from the initial guess of the treatment plan with new optimized weightings.

### 2.4 ROWD optimized cases and hypoxia estimates

The optimization tool was applied on a simulated water phantom and on two HNC patients. Initial treatment plans were first created in the Eclipse TPS (Varian Medical Systems, Palo Alto, California, US) with a constant RBE of 1.1 (RBE_1.1_). The treatment plans were then re‐optimized in our MC based treatment planning tool, with ROWD based on an RBE of 1.1 (RBE_1.1_(OER)), RBE from ROR (ROR(OER)), RBE from MCN (MCN(OER)), and the reference RBE_1.1_ without OER effects (RBE_1.1_). The Eclipse plans and re‐optimized plans were recalculated in the FLUKA MC code, with number of simulated primary particles chosen to give a voxel mean statistical uncertainty <2%.

**FIGURE 1:**
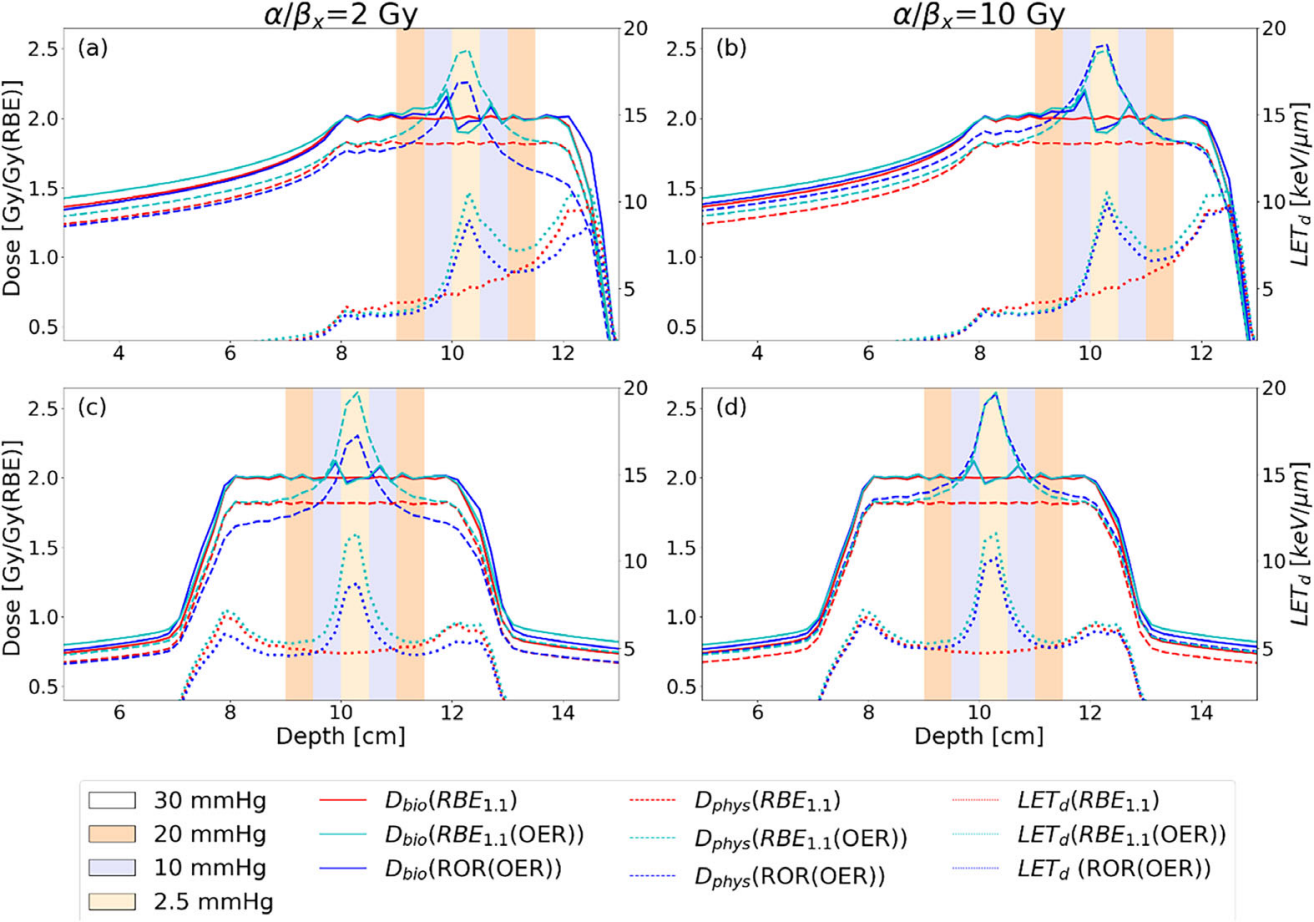
Spread‐out Bragg peak in water with a single field (a, b) and two opposing fields (c, d), optimized to the ROR(OER) model and RBE_1.1_ models (RBE_1.1_ and to RBE_1.1_(OER)) applying the Monte Carlo based optimizer. The physical dose for the respective models are given as dashed lines and the ROWD are given as dashed lines, and the linear energy transfer (LET) as dotted lines. OER, oxygen enhancement ratio; RBE, relative biological effectiveness; ROR, Rørvik model; ROWD, RBE and OER weighted dose.

Different hypoxia levels were simulated for a water phantom by dividing it into seven parts in the beam direction. Each part had different pO_2_ values, between 2.5 and 30 mmHg, as illustrated in Figure 1. Two treatment plans were initially created in Eclipse for the water phantom with a 4 × 4 × 4 cm^3^ target at a depth of 8 cm: a single field spread‐out Bragg peak (SOBP) and a SOBP with two opposing fields, both with a RBE_1.1_ dose of 2 Gy(RBE). Re‐optimization and recalculation were done with scoring voxels of 2 × 2 × 2 mm^3^ for both the single field SOBP and opposing fields SOBP.

The treatment plans of the patients were originally generated in Eclipse to a RBE_1.1_ target dose of 70 Gy(RBE) delivered with three treatment fields in 35 fractions. When re‐optimizing the treatment plans, constraints were put on the left parotid glands to keep the dose lower than the prescribed dose, as it was the OAR closest to the PTV. The high max dose constraint (70 Gy(RBE)) Although there are differences, the similarity for the parotis comes from the overlapping parts to the PTV, and the difference in ${\left(\alpha /\beta \right)}_{x}$ as the values were 10 Gy to the PTV^24^ while for the OAR, an ${\left(\alpha /\beta \right)}_{x}$ of 3 Gy was used,^25^ causing a higher estimated RBE in the parotis compared to the PTV.

## 3 RESULTS

### 3.1 Water phantom

ROWD optimization resulted in a homogeneous ROWD across the SOBP, but with distinctly higher LET_d_ and physical dose for the hypoxic regions in the SOBP compared to RBE_1.1_‐based optimization (Figure 1). In RBE_1.1_(OER)‐based optimization, this led to maximum physical doses of 2.5 and 2.6 Gy, for the single and opposing field plans, respectively, in the most hypoxic areas. In comparison, the maximum physical dose from the ROR(OER) in the most hypoxic were 2.3 and 2.5 Gy for two fields, for ${\left(\alpha /\beta \right)}_{x}$ = 2 and 10 Gy, respectively. The results for the MCN plans were similar and the results can be found in the Supplementary Materials.

The LET distribution from the ROWD optimized plans differed greatly from the conventional RBE_1.1_, as the LET was elevated in the most hypoxic areas. The RBE_1.1_(OER) model provided the highest LET values with peaks of 10 and 12 keV/μm for the single field and two‐fields plans, respectively. Similarly, the LET peaks for the ROR(OER) model were 10 keV/μm for both single field and the two‐field plans, respectively.

### 3.2 Head and neck cancers

**FIGURE 2:**
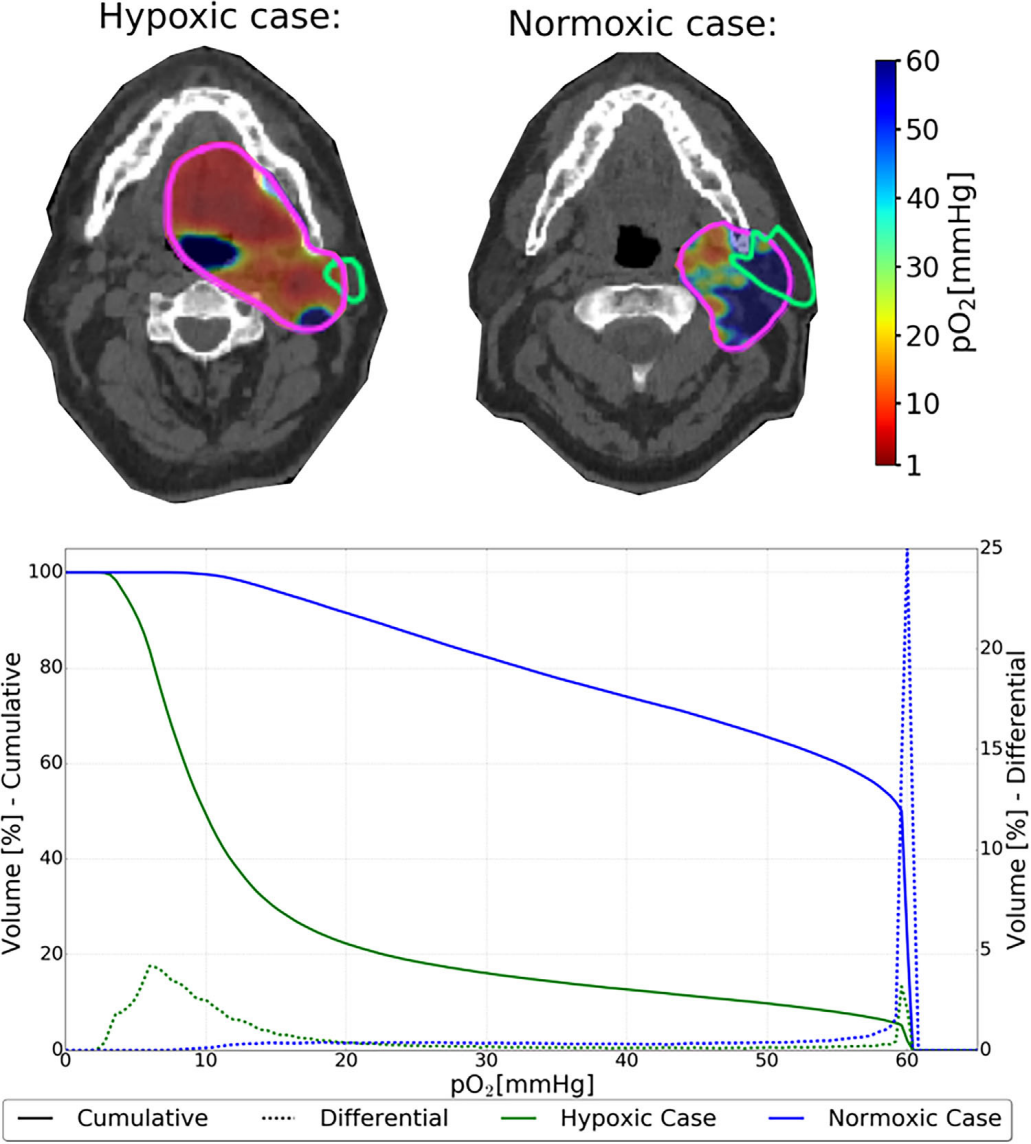
The partial oxygen pressure (pO_2_) distribution for the two HNC cases. The top row shows a slice of each patient with the pO_2_ values plotted on top. The bottom plot show histograms of the pO_2_ values for the PTV in the two cases.

For the two HNC patients, one case had overall lower pO_2_ (denoted hypoxic case) compared to the other case (denoted normoxic case), and they thus represent two different clinical scenarios. This is visualized in Figure 2, where a colorwash plot of the pO_2_ distributions can be seen, as well as pO_2_ volume histograms. It shows that the pO_2_ values are very different for the two cases. Regions with pO_2_ values above 60 mmHg are considered as normoxic (OER equal to 1). For the hypoxic case, 95% of the PTV had pO_2_ values below this threshold (values below 60 mmHg), while for the normoxic case, the corresponding value was 50%.

**FIGURE 3:**
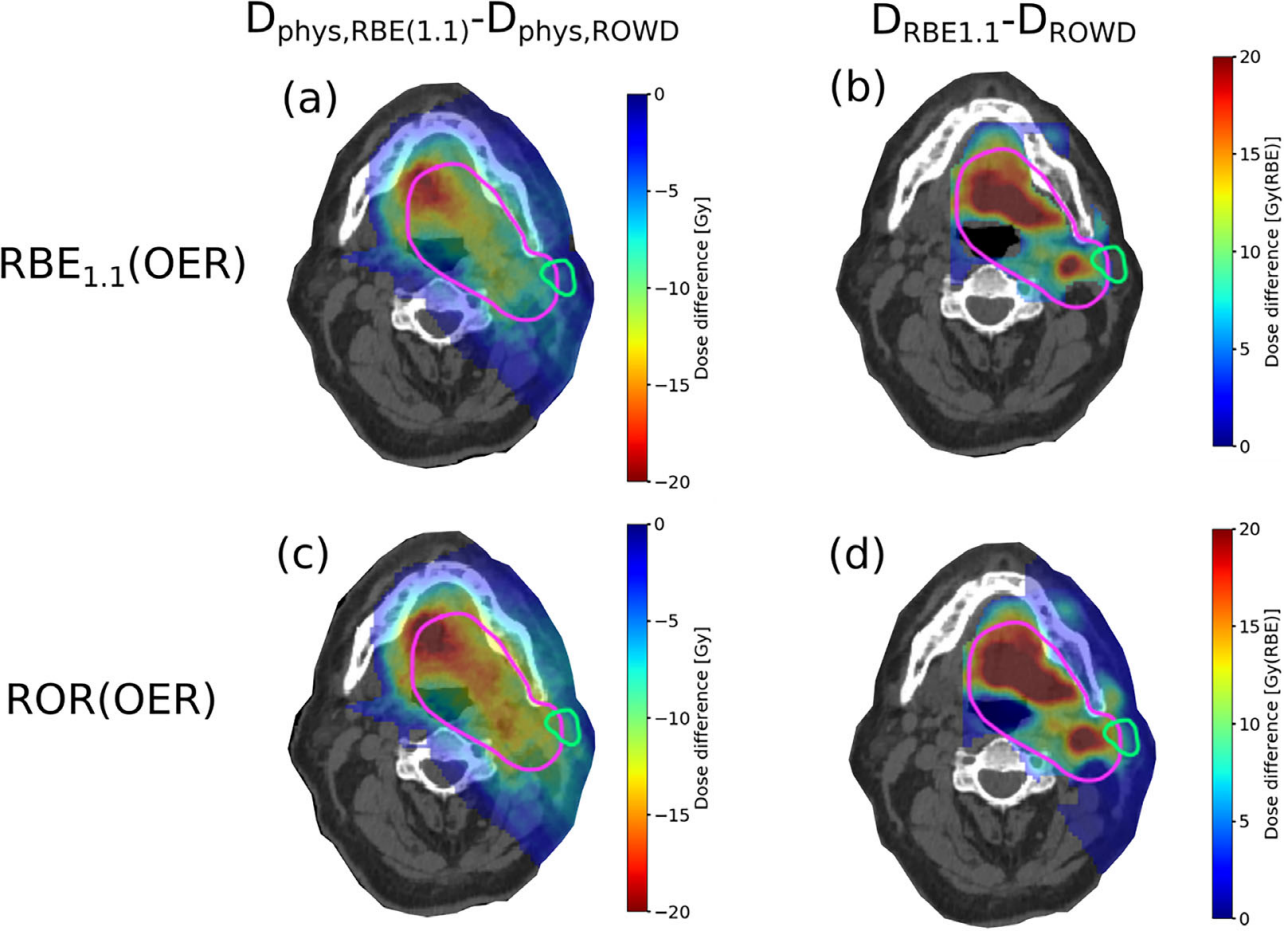
The difference in physical dose between the RBE_1.1_ reference plan and the different ROWD optimized plans (a, c), and the difference between RBE_1.1_ dose and ROWD for the different ROWD optimized plans (b, d) in the hypoxic case. The PTV is delineated in pink and the left parotid gland in green. OER, oxygen enhancement ratio; RBE, relative biological effectiveness; ROR, Rørvik model; ROWD, RBE and OER weighted dose.

**FIGURE 4:**
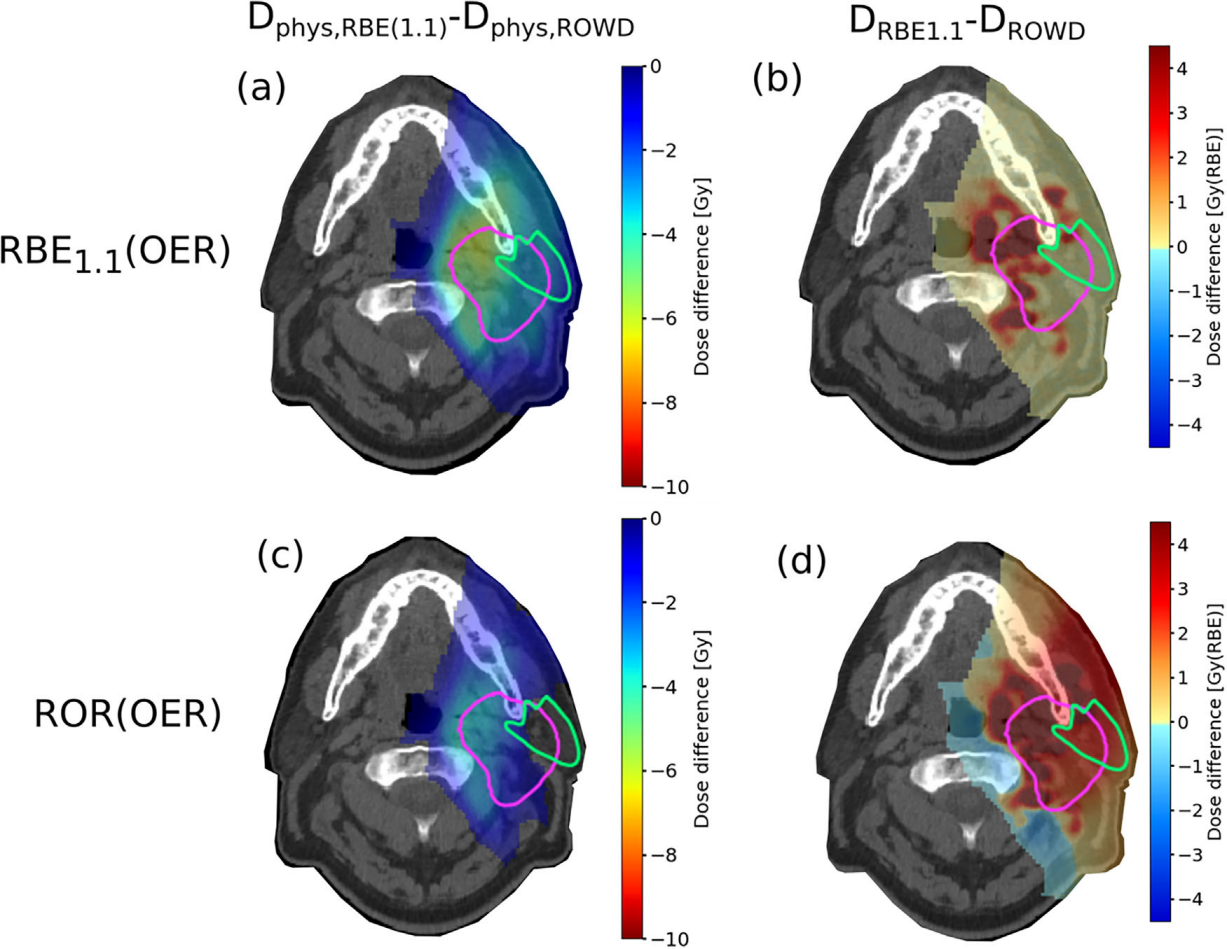
The difference in physical dose between the RBE_1.1_ reference plan and the different ROWD optimized plans (a, c), and the difference between RBE_1.1_ dose and ROWD for the different ROWD optimized plans (b, d) in the normoxic case. The PTV is delineated in pink and the left parotid gland in green. OER, oxygen enhancement ratio; RBE, relative biological effectiveness; ROR, Rørvik model; ROWD, RBE and OER weighted dose.

A median (*D*_50%_) PTV dose of 70 Gy(RBE) was achieved for both cases which agrees well with the prescribed dose. For the hypoxic case, the severely hypoxic regions of the tumor resulted in a significant elevation of the physical dose for the ROWD optimized plan, with an up to 19% increase mean dose, compared to the RBE_1.1_ reference plan, as seen in the left column of Figure 3. The regions with the largest difference in physical dose between the RBE_1.1_ reference plan and the ROWD optimized plan correlated well with the areas with the low pO_2_ values in the hypoxic case (Figure 2). Consequently, a greater difference between the RBE_1.1_ reference plan and the ROWD optimized plans was seen for the hypoxic case compared to the normoxic case (Figures 3 and 4). Still the dose difference plot in Figure 4 indicates that the optimization takes the pO_2_ distribution (Figure 2) into account also for the normoxic case.

**FIGURE 5:**
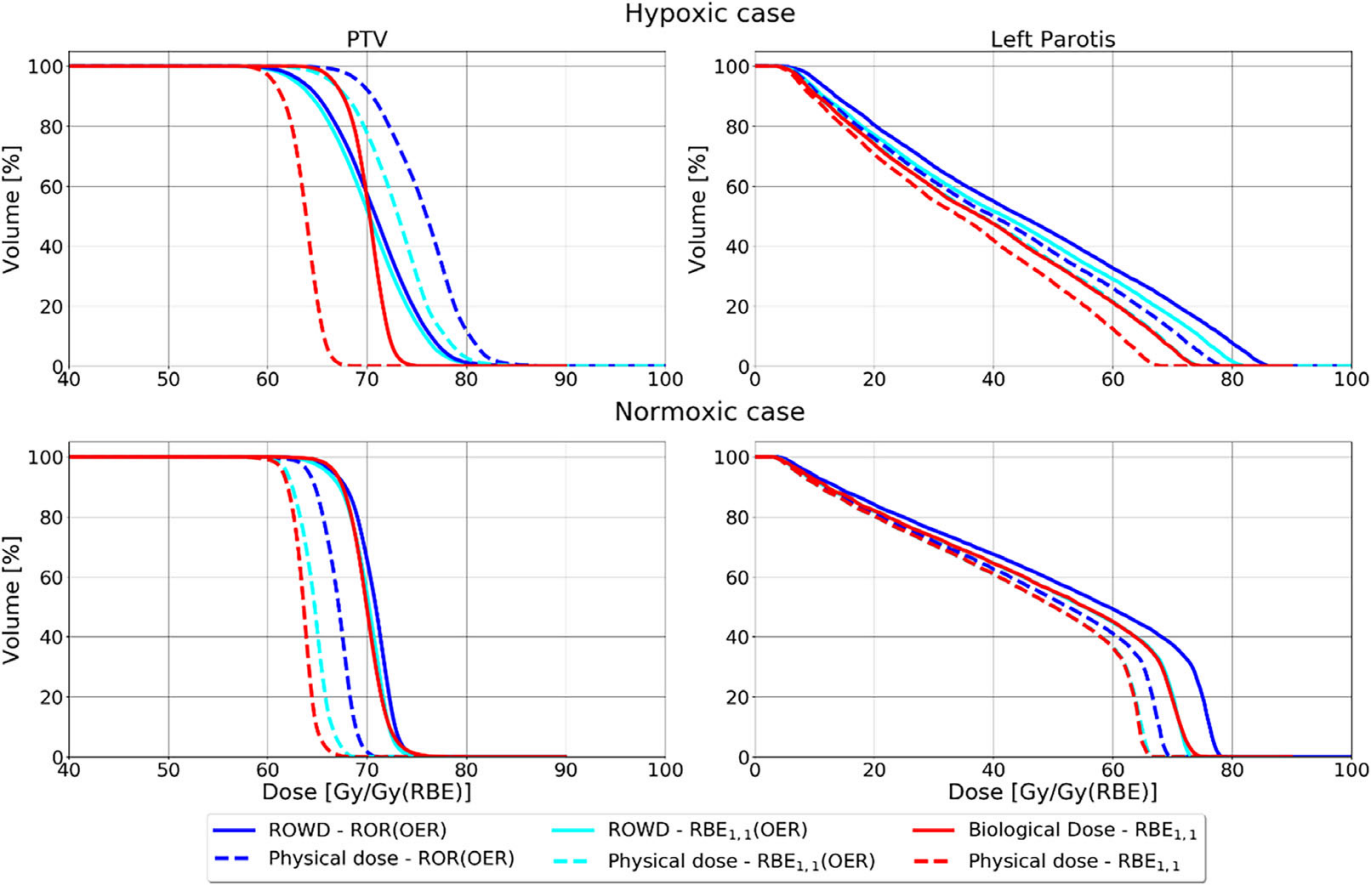
DVHs for the different plans for the hypoxic case (top row) and the normoxic case (bottom row) where the solid lines represent the ROWD color coded to each RBE model, while the dashed lines represent the respective physical dose using the same color coding for RBE models. OER, oxygen enhancement ratio; PTV, planning target volume; RBE, relative biological effectiveness; ROR, Rørvik model; ROWD, RBE and OER weighted dose.

The impact of applying ROWD optimization was strongly dependent on the level of hypoxia. In the hypoxic case (Figure 5), the physical doses were significantly higher for the RBE_1.1_ plan compared to the ROWD optimized plans. Differently, the physical doses for the ROWD‐optimized plans for the normoxic case were more similar to the RBE_1.1_ reference plan. The mean physical dose to the PTV was 12.2 Gy higher for the ROR(OER) optimized plan compared to the RBE_1.1_ plan, and 9.3 Gy higher for the RBE_1.1_(OER) plan for the hypoxic case. For the normoxic case, the ROR(OER) plan provided a mean physical dose 3.5 Gy higher compared to the reference plan, and 1.1 Gy higher for the RBE_1.1_(OER) plan.

**FIGURE 6:**
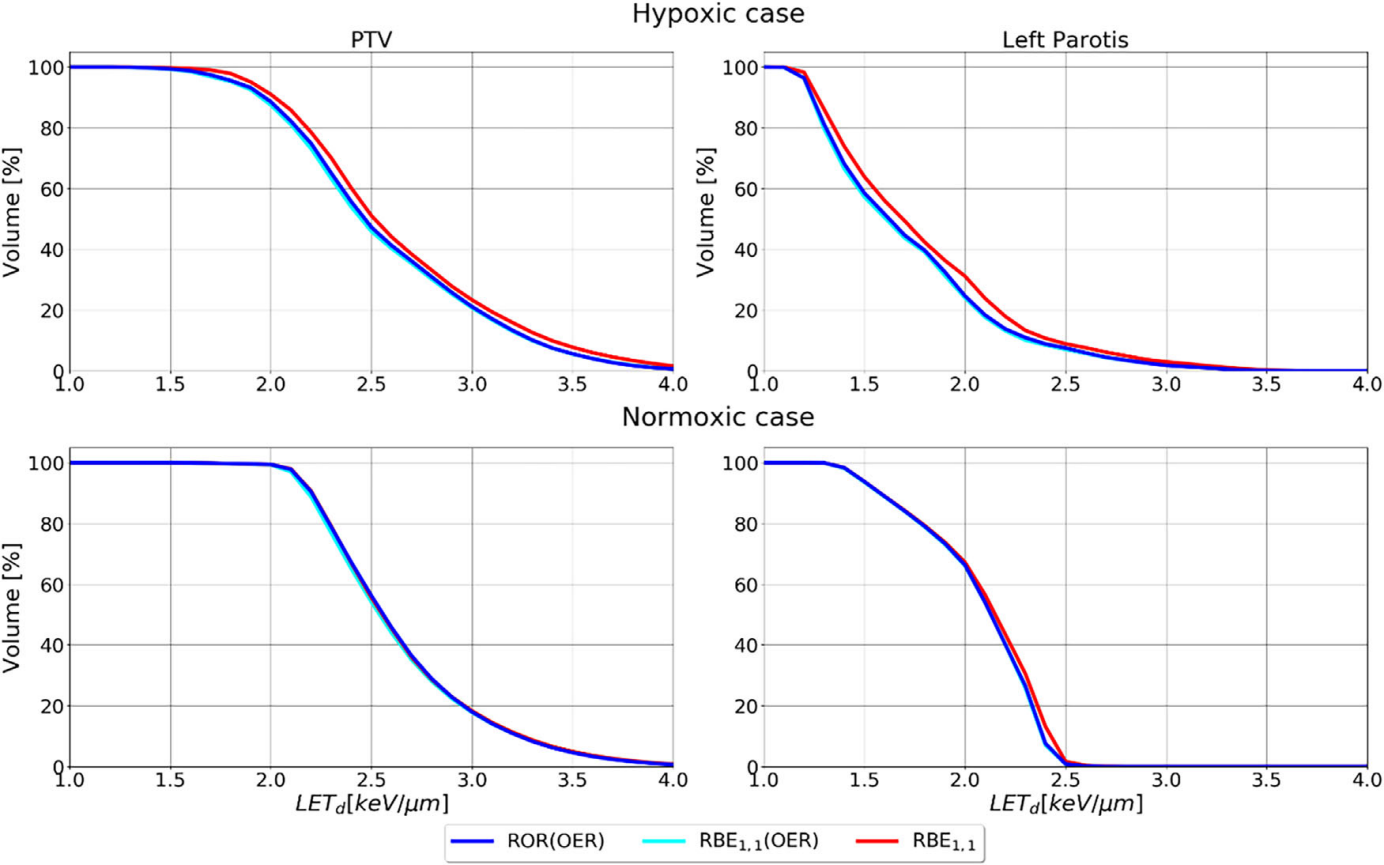
LET volume histogram for the hypoxic (top row) and normoxic case (bottom row). OER, oxygen enhancement ratio; PTV, planning target volume; RBE, relative biological effectiveness; ROR, Rørvik model.

For the OARs, the ROWD‐optimized plans frequently resulted in a higher physical dose compared to the RBE_1.1_ reference plan as seen in the DVHs in Figure 5. The physical dose differences between the reference plan and ROWD optimized plans, were smaller for the normoxic case, and higher for the hypoxic case. For the hypoxic case, the maximum physical dose to the OAR increased by 10.7 Gy from the ROR(OER) optimized plan, compared to the reference plan, while for the RBE_1.1_(OER) plan, the increase was 6.7 Gy. For the normoxic case the increase in maximum physical dose from the ROR(OER) optimized plan and the reference plan were below 3.3 Gy. However, we can see from Figure 4a,c that there are still dose differences in some regions. For the RBE_1.1_(OER) optimized plan, the maximum physical dose was slightly increased compared to the reference plan. In the hypoxic case, the mean LET in both the PTV and the OAR was lower for the different ROWD‐optimized plans compared to the RBE_1.1_ reference plan, as seen in the LET volume histogram in Figure 6, while no significant difference was found for the normoxic case.

## 4 DISCUSSION

In this study, we implemented a method for including hypoxia in voxel‐by‐voxel RBE and OER weighted dose optimization. The implementation was demonstrated on a simulated water phantom with well‐defined regions of varying oxygen levels. Then, clinical proof of concept was evaluated in two HNC cases where voxel‐wise oxygen levels were estimated from [^18^F]‐EF5 PET images. As intended, the optimized proton plans resulted in increased physical dose in areas with low oxygen levels. The LET distributions for the clinical cases were, however, little affected by the ROWD optimization. The median target ROWD also corresponded well with the prescription dose. While the method was created for protons, it can also be applied for heavier ions like helium and carbon.

The water phantom case, optimized to account for the phantom oxygen levels, showed nearly homogeneous ROWD to the target volume. Exceptions were seen close to the borders between the different oxygen levels, were the ROWD was more heterogenous. A completely homogeneous ROWD would not have been physically possible, as it would have required a perfectly rectangular physical dose distribution. The ROWD and physical dose outside the target volume for the RBE_1.1_(OER) plans was also higher for the treatment plans optimized to hypoxia, compared to the *D*_RBE1.1_ plan. This shows that dose escalation based on hypoxic regions might violate current normal tissue constraints. However, dose escalation based on hypoxic regions may also reduce the risk of recurrence in the PTV which again can result in a reduced probability of having to re‐irradiate the patient which could have increased the dose to the OARs even more. This is also supported by a study by Wright et al.^26^ which shows that [^18^F]‐EF5 PET images have high repeatability showing that the current optimized plan will be sufficient in terms of image robustness. In the water phantom, the increased LET contributed to the ROWD in almost the same extent as the elevation of physical dose, as it in the most hypoxic area was almost doubled compared to the RBE_1.1_ plan, along with the physical dose also significantly higher than the RBE_1.1_‐plan. This shows the potential of ROWD based optimization, where the LET would contribute to achieve high enough ROWD to the tumor to account for highly hypoxic areas. This is also shown by Mein et al.^27^ as they demonstrated that through arc therapy and achieving higher LET in a target could improve treatment outcome for hypoxic tumors.

The tool was further explored in two HNC patient cases, where a reasonably homogeneous PTV ROWD was observed when accounting for hypoxia. For the hypoxic case, an increased ROWD to the left parotid gland was observed. Too high dose to the parotids can result in impaired gland function leading to xerostomia (dry mouth), which will reduce the patient's quality of life following treatment.^28^ However, studies have suggested that sparing at least one parotid gland to a mean dose of 20 Gy seems to eliminate xerostomia,^28^ and this was achieved in our HNC cases. For the normoxic case there were smaller differences between the ROWD optimized plans and the reference plan, however, as there were indications of similarities between the pO_2_ maps and the difference between ROWD and RBE_1.1_ dose, it indicates that although the impact of ROWD optimization is smaller, it could still contribute to a better treatment outcome. The RBE_1.1_(OER) optimized plan showed a lower physical dose distribution compared to the ROR(OER) optimized plan, indicating that even though the OER is used together with RBE_1.1_, it is still underestimating the ROWD, as the ROR model predicts a higher ROWD due to the ${\left(\alpha /\beta \right)}_{x}$ dependency and the high ${\left(\alpha /\beta \right)}_{x}$ in the tumor. Although there are differences, previous studies^14^ have shown similar dose distribution between variable RBE models and RBE_1.1_ when the ${\left(\alpha /\beta \right)}_{x}$ is high, which is also seen in this study, as the OER and RBE is independent of each other, the RBE term will be affected by the ${\left(\alpha /\beta \right)}_{x}$. In vitro based RBE models such as the ROR and MCN model are also influenced by the uncertainties and differences in the cell‐survival experiments.^29^ There is also considerable uncertainty in the translation of in vitro results to clinical endpoints. These uncertainties need to be addressed before clinical RBE optimization is broadly adapted. The difference between the reference plan and the ROWD plan in terms of tumor coverage implies that the pO_2_ map is the reason for the suboptimal treatment, as it is not possible to achieve a perfect dose distribution between neighboring voxels with large difference in pO_2_. This is supported by the sensitivity analysis of the ROWD and OER found in the Supplementary Materials.

Further, for the two HNC cases, similar to the water phantom case, the ROWD was mostly homogenous, but in regions with a large difference in pO_2_ values, the physical dose distribution become insufficient to cover the range of physical dose. It could be possible to achieve an overall more homogenous dose distribution by increasing the number of pencil beams in each field (e.g., by reducing the spot spacing), especially in the hypoxic areas, to enhance the LET values in the tumor, reducing heterogeneity in physical dose in regions with large differences in pO_2_. This could also be achieved by using opposing fields, or arcs, which will make it easier to manipulate the ROWD. Another method could be to add an increased dose objective for areas with low pO_2_ values, which might overdose some parts of the tumor, but increase the overall dose conformity.

However, McKeown et al.^30^ defined pathological hypoxia at 8 mmHg, and found HNC cases to have a median tumor pO_2_ between 10.0 and 14.7 mmHg. In this study, we saw that approximately 50% of the tumor in the hypoxic case in this case had estimated pO_2_ values equal to or under this limit, reflecting the similarity to other HNC cases.

The LET distribution in the HNC cases were almost similar for all strategies (RBE_1.1_ slightly higher, as seen in Figure 6), opposed to the water phantom case where the LET values differed greatly from the reference RBE_1.1_ plan. This could be because of the pO_2_ maps, as the map for the water phantom case has the hypoxic region in the middle of the target, while for the first HNC case, most of the tumor was hypoxic, with only small regions being non‐hypoxic. This leads to a difficulty of elevating the LET in certain areas, as hypoxic hotspots are needed to elevate the LET, as illustrated in the water phantom, while there are cold spots instead in the hypoxic case. In the normoxic case, the pO_2_ values are generally high, with small hotspots. Although there were no increase in the LET for the clinical cases used in this study, LET increase in certain regions could be achieved if this is prioritized higher in the optimization process. The elevations in the middle region of the water phantom case suggest that the LET could also be a large factor in providing a better treatment for HNC patients with more suitable pO_2_ maps, as also suggested with several LET painting studies.^2^, ^4^ However, the overall low pO_2_ values in the tumor volume, with small cold spots (high pO_2_), could be the reason for these lower LET values, as the optimizer will try to reduce the ROWD in areas where the pO_2_ is low, and in this case, reducing the LET. A study by Malinen and Sovik^2^ also showed a larger effect from dose painting than with LET painting with protons, suggesting that physical dose manipulation provides the best treatment outcome. However, identifying the potential for LET painting in patients with smaller hypoxic regions could still be of interest as it is possible to reach higher LET values for small subvolumes compared to the relatively large hypoxic volume in the hypoxic case in the present study. Some reduction in the OER combined with an increase in the (aerobic) RBE could give a substantial effect for high LET values, as illustrated in Figure A1b) and A1d) in the Supplementary Materials. Still, uncertainties are surely considerable in the fitting of the OER versus LET for protons as the amount of available experimental data is limited.

Estimation of pO_2_ in a patient is associated with uncertainties.^31^ This was observed in our previous study,^17^ where we introduced the method for estimating pO_2_ from [^18^F]‐EF5 PET images. However, the pO_2_ was only estimated in the tumor, and it was seen from the PET data applied in the PET uptake to pO_2_ conversion curve that it can be expected that the method underestimates hypoxia.^17^ Thus, while the treatment plans optimized in this study may underestimate the physical dose which should be prescribed to the tumor area, the optimization would be on the safe side for normal tissue. Still, to fully explore the potential of hypoxia adapted treatment planning, reliable estimates of the pO_2_ is necessary. In order to achieve this, comparisons between pO_2_ estimations based on different imaging modalities which can depict hypoxia may be useful, for example, comparisons of different hypoxia PET tracers or magnetic resonance imaging (MRI) techniques. PET imaging is currently the preferred method of imaging tumor hypoxia,^32^ and different methods for estimating pO_2_ from PET uptake have been proposed.^33^, ^34^ However, several methods for depicting hypoxia with MRI exists also.^35^ Ideally, these methods should also be validated by demonstrating a correlation between the estimated pO_2_ distribution to quantification of hypoxia in excised tumor tissue, for instance by immunohistochemical staining of tissue sections.

Several methods of overcoming tumor hypoxia have been proposed.^5^ This includes kill painting, based verified also with cell survival experiments on extended targets,^7^, ^11^ and LET painting, which have shown potential for increased tumor control probability with protons.^2^ The ROWD method applied in this study, where we optimize hypoxic tumors on a voxel‐by‐voxel basis with RBE effects taken into account, has the benefit over dose painting and LET painting by the inclusion of a variable RBE in addition to the OER in the optimization. LET‐guided optimization is generally not yet available for clinics, however, most proton centers check for overlap between high dose and high LET.^36^ This method is an alternative simplified version of the latter, with a different OER model (Wenzl & Wilkens^18^ with a subset of only proton data) and phenomenological RBE models instead of the LEMIV used there. Both techniques would benefit from more in vitro data between hypoxic and normoxic cells. We also included clinical relevant hypoxia data from EF5‐PET images. Also, with improved availability of hypoxia imaging, our method could become more clinical relevant in the future. While our method is tailored for proton therapy, we also anticipate that it can be used with other ions, including helium and carbon, also potentially including different RBE models, beyond the ones used in the present study.

## 5 CONCLUSION

We have implemented and explored a ROWD optimization method which accounts for hypoxia in a FLUKA MC based treatment planning tool. The implementation was successful, with median target ROWD corresponding to the prescription dose and increased physical dose in hypoxic regions. Including OER in dose optimization for simple SOBP scenarios led to a large increase in both physical dose and LET in the hypoxic regions. The increase in LET was reduced when combining the OER model with RBE models, thus the choice of model can affect both the LET and physical dose distribution from ROWD optimization in proton therapy. Comparing the SOBP results to the patient plan results shows that the change in LET and physical dose from ROWD optimization most likely is strongly dependent on the location and size of the hypoxic region.

## AUTHOR CONTRIBUTIONS

All corresponding authors have contributed directly to the intellectual content of this manuscript. Tordis Johnsen Dahle, Sara Pilskog, Andrea Mairani, Camilla Stokkevåg, and Kristian Smeland Ytre‐Hauge suggested the idea for this study and presented the theory and basis for the method. Heikki Minn were responsible for the hypoxia‐data and the methods regarding the imaging of hypoxia, and Kathrine Røe Redalen and Eirik Malinen were responsible of the analysis of the hypoxia‐data. Camilla Grindeland Boer was responsible for the treatment planning performed in this study, and Camilla Grindeland Boer, Sara Pilskog, and Camilla Stokkevåg contributed to the clinical analysis of the treatment planning. Helge Henjum was responsible for performing the method and analysis of the data, and the main author. All authors contributed to the discussion of the results.

## CONFLICT OF INTEREST STATEMENT

The authors declare no conflicts of interest.

## Supporting information

Supporting information

## Data Availability

All data generated and analyzed during this study are included in this published article (and its supplementary information files).
