# Supplementary material for: Combined RBE and OER optimization in proton therapy with FLUKA based on EF5‐PET
Source: J Appl Clin Med Phys. 2023 May 10;24(9):e14014. doi: 10.1002/acm2.14014 (PMC10476997; doi:10.1002/acm2.14014)
Supplement: Supplementary file 1 — Supporting information [file ACM2-24-e14014-s001.docx]

Supplementary Material

#### RBE models

The variable RBE models are decided by the parameters $RBE_{max}$ and $RBE_{min}$, defined as

$$\lim_{D_{p}\to0}RBE=RBE_{max} =\frac{\alpha}{\alpha_{x}}$$

$$\lim_{D_{p}\to\infty}RBE=RBE_{min} =\sqrt{\frac{\beta}{\beta_{x}}}.$$

In our study we use the

**MCN model**

The MCN model is based on a regression fit to 287 experimental data points. It is based on all published *in vitro* cell survival data [22]. The $RBE_{max}$ and $RBE_{min}$ are defined as follows:

$$RBE_{max}=0.99064 +\frac{0.35605 Gy}{\left( \frac{\alpha}{\beta} \right)_{x}}LET_{D}$$

$$RBE_{min}=1.1012- 0.0038703Gy^{-\frac{1}{2}}\left( keV \mu m \right)^{-1}\sqrt{\left( \frac{\alpha}{\beta} \right)_{x}}LET_{D}$$

**ROR model**

ROR is based on a non-linear dependency between the RBE and LET. The $\mathrm{RBE}_{\min} = 1, while the {RBE}_{max}$ is based on a biological weighting function (BWF) which is derived from *in vitro* cell experiments. The BWF $(r_{max}\left( L \right))$ weights the LET spectrum $(d\left( L \right))$. The $RBE_{max}$ and $RBE_{min}$ are defined as follows:

$${RBE}_{max}\left( d(L) \right)=\int_{0}^{\infty} r_{max}\left( L \right)d\left( L \right)dL,$$

$$r_{max}\left( L \right)=1+\frac{1 Gy}{{(\alpha}/{{\beta)}_{x}}}\left( 0.578\left( \frac{\mathrm{keV}}{\mu m} \right)^{-1}L-0.0808\left( \frac{\mathrm{keV}}{\mu m} \right)^{-2}L^{2}+0.00564\left( \frac{\mathrm{keV}}{\mu m} \right)^{-3}L^{3}-9.92\times{10}^{-5}{\left( \frac{\mathrm{keV}}{\mu m} \right)^{-4}L}^{4} \right), L<37.0\frac{\mathrm{keV}}{\mu m}$$

$$r_{max}\left( L \right)=1+10.5\frac{Gy}{\left( \alpha/\beta\right)_{x}} L\geq37.0\frac{\mathrm{keV}}{\mu m}$$

$${RBE}_{min}=1$$

#### Cost function

For the optimization problem in this study, we defined the cost function to be minimized as:

|  | $\chi^{2}\left( \boldsymbol{N} \right)=\sum_{j\epsilon PTV} \frac{w_{j}\left( {\hat{\boldsymbol{D}}}_{j}-\boldsymbol{D}_{j} \right)^{2}}{{\hat{\boldsymbol{D}}}_{j}^{2}}+\sum_{j\epsilon OAR} \frac{w_{j}\left( {\hat{\boldsymbol{D}}}_{j}-\boldsymbol{D}_{j} \right)^{2}}{{\hat{\boldsymbol{D}}}_{j}^{2}}\Theta\left( {\hat{\boldsymbol{D}}}_{j}-\boldsymbol{D}_{j} \right),$  . |  |
| --- | --- | --- |

where ${\hat{\boldsymbol{D}}}_{j}$ is the prescribed dose in voxel $j$, $\boldsymbol{D}_{j}$ is the dose in voxel *j,* and $w_{j}$ is the weighting factor for the different PTV and OARs. $\Theta$ is the Heaviside function, with contribution only if the dose in the voxel is higher than the prescribed dose.

#### Additional plots

***Sensitivity study***

In Figure A1***,*** a sensitivity analysis of the OER and ROWD when varying the different parameters can be seen.


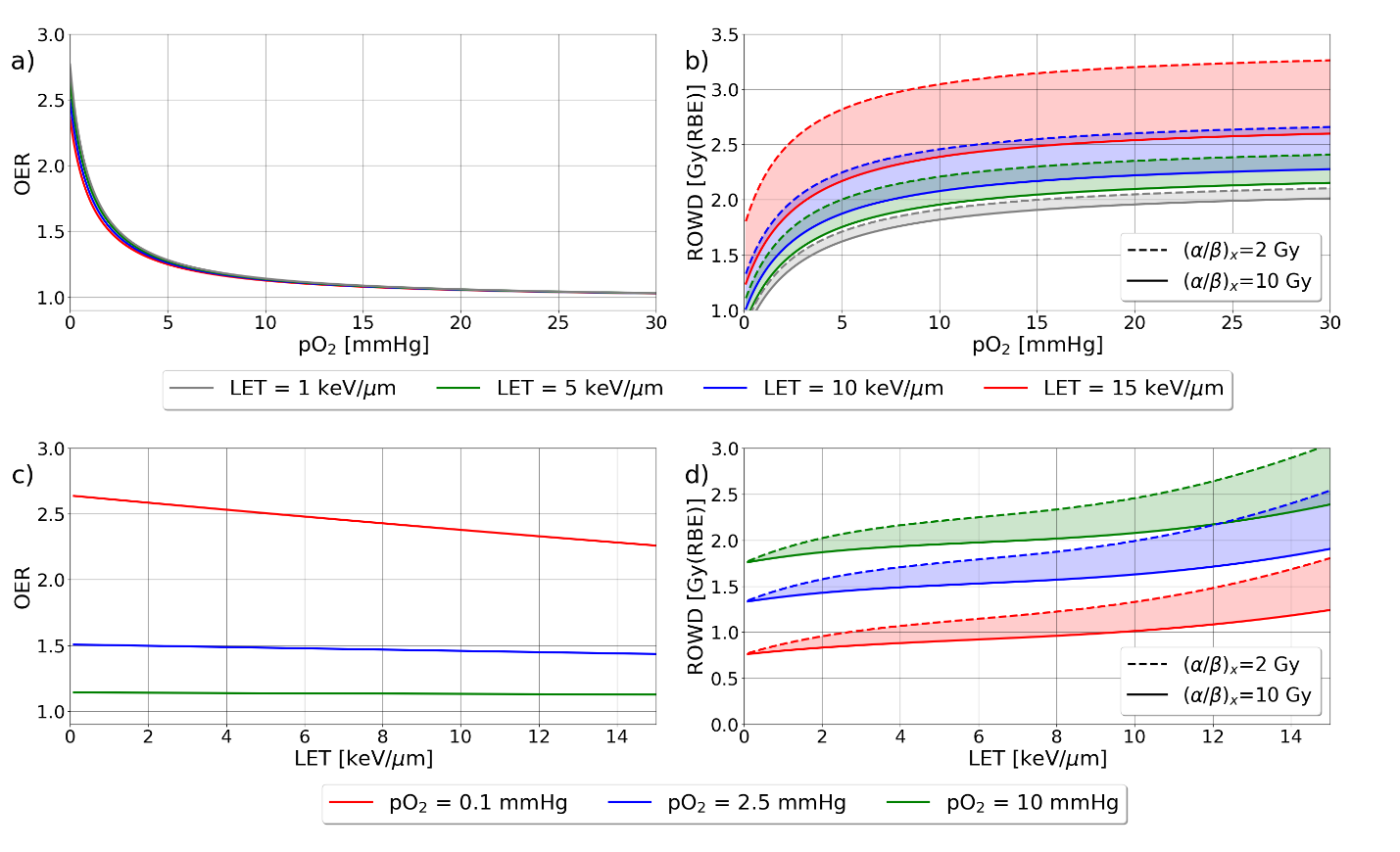


Figure A1 a) OER as a function of pO_2_ for different values of LET, b) ROWD as a function of pO_2_ for different values of LET, with a physical dose of 2 Gy and two different $\left( \alpha/\beta\right)_{x}$calculated with the ROR model, c) OER as a function of LET for different pO_2_ values and d) ROWD as a function of LET for different pO_2_ with a physical dose of 2 Gy and two different $\left( \alpha/\beta\right)_{x}$, calculated with the ROR model.

*Water Phantom*

In this chapter, we present additional plots for this study in terms of depth dose curve for the MCN(OER)-optimized plan in the water phantom (Figure A2), and the DVH for the water phantom case (Figure A3).


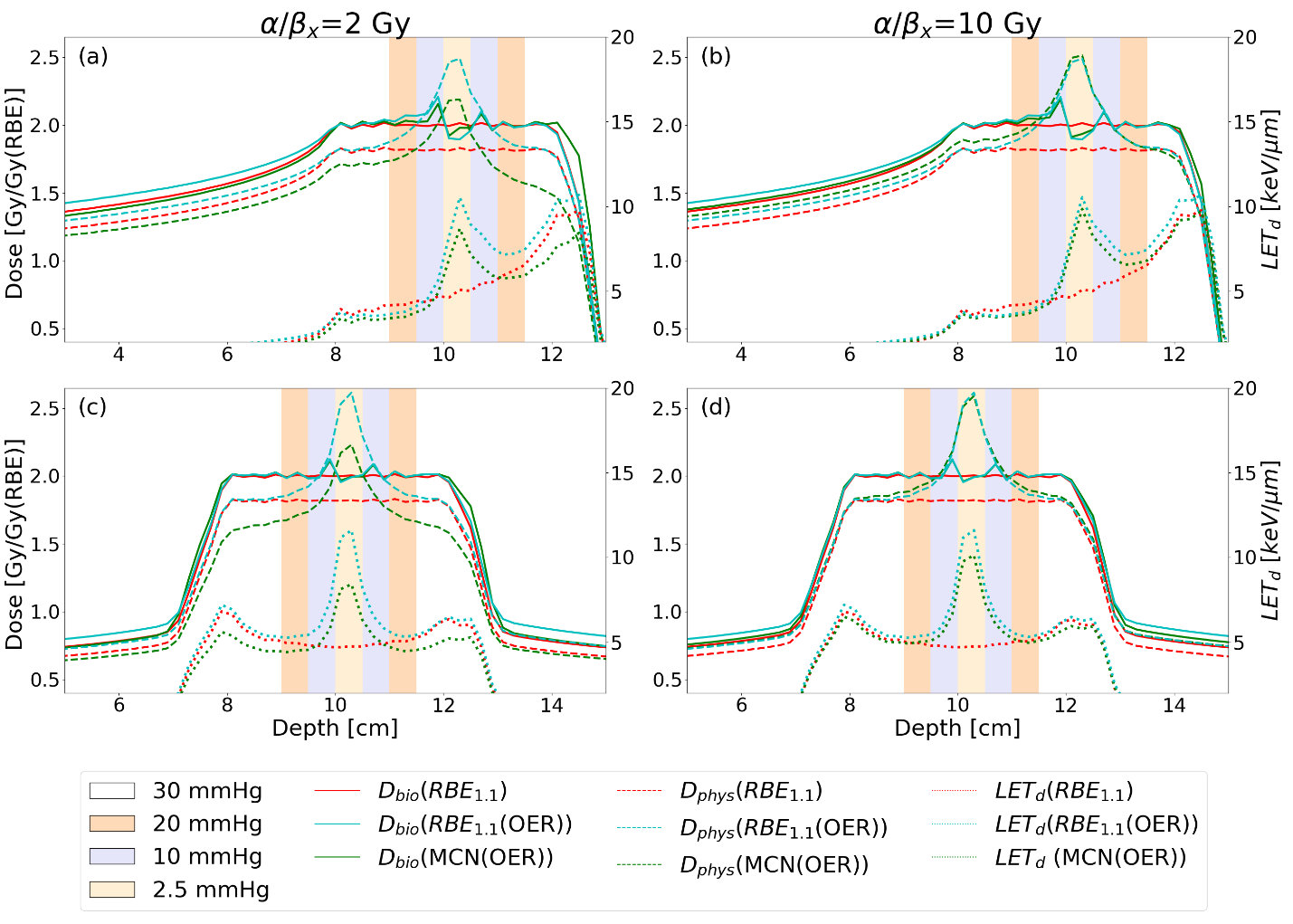


Figure A2 Spread-out Bragg peak in water with a single field (a and b) and two opposing fields (c and d), optimized to the MCN(OER) model and RBE_1.1_ models (RBE_1.1_ and to RBE_1.1_(OER)) applying the Monte Carlo based optimizer. The physical dose for the respective models are given as dashed lines and the ROWD are given as dashed lines, and the LET as dotted lines.


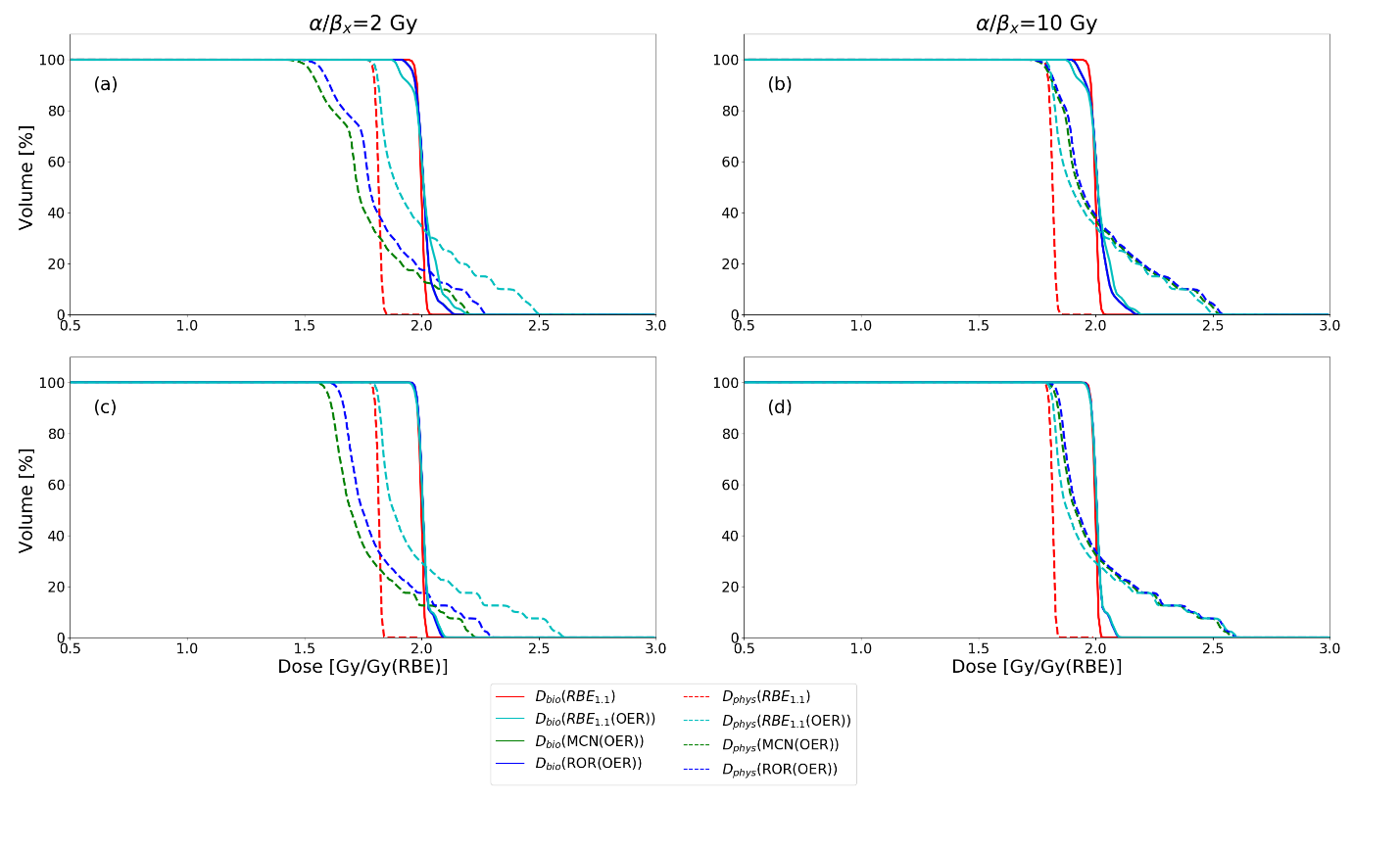


Figure A3 DVHs for the water phantom case with a single field (a and b) and two fields (c and d). The physical dose for the respective models are given as solid lines, while the RBE-weighted dose are given as dashed lines.

*HNC cases*

In Figure A4 we see the difference physical dose between the MCN(OER) optimized plan, and the reference plan (a and c), and the difference in RBE_1.1_-weighted dose and ROWD for the MCN(OER) optimized plan (b and d). We also see the DVH for the MCN(OER)-optimized plan (Figure A5), along with the RBE_1.1_(OER) optimized plan and RBE_1.1_ reference plan, for comparison. Similarly, we show a LET volume histogram for MCN(OER) in Figure A6, along with the RBE_1.1_(OER) optimized plan and RBE_1.1_ reference plan, for comparison.


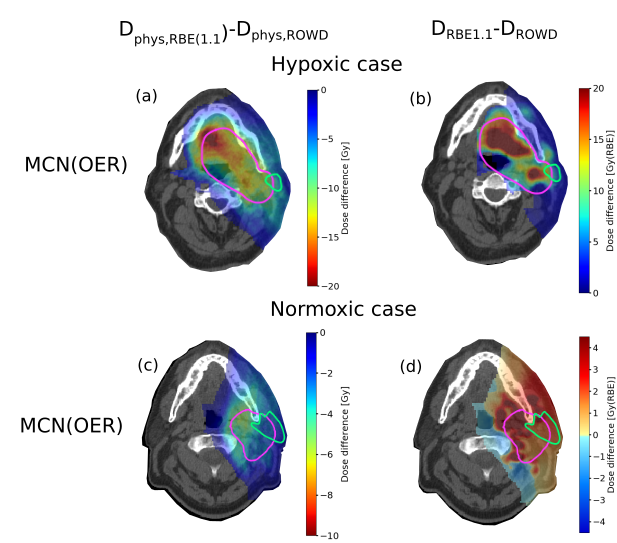


Figure A4 The difference in physical dose between the RBE_1.1_ reference plan and the different ROWD optimized plans (a and c), and the difference between RBE_1.1_ dose and ROWD for the different ROWD optimized plans (b and d) in the hypoxic case. The PTV is delineated in pink and the left parotid gland in green.


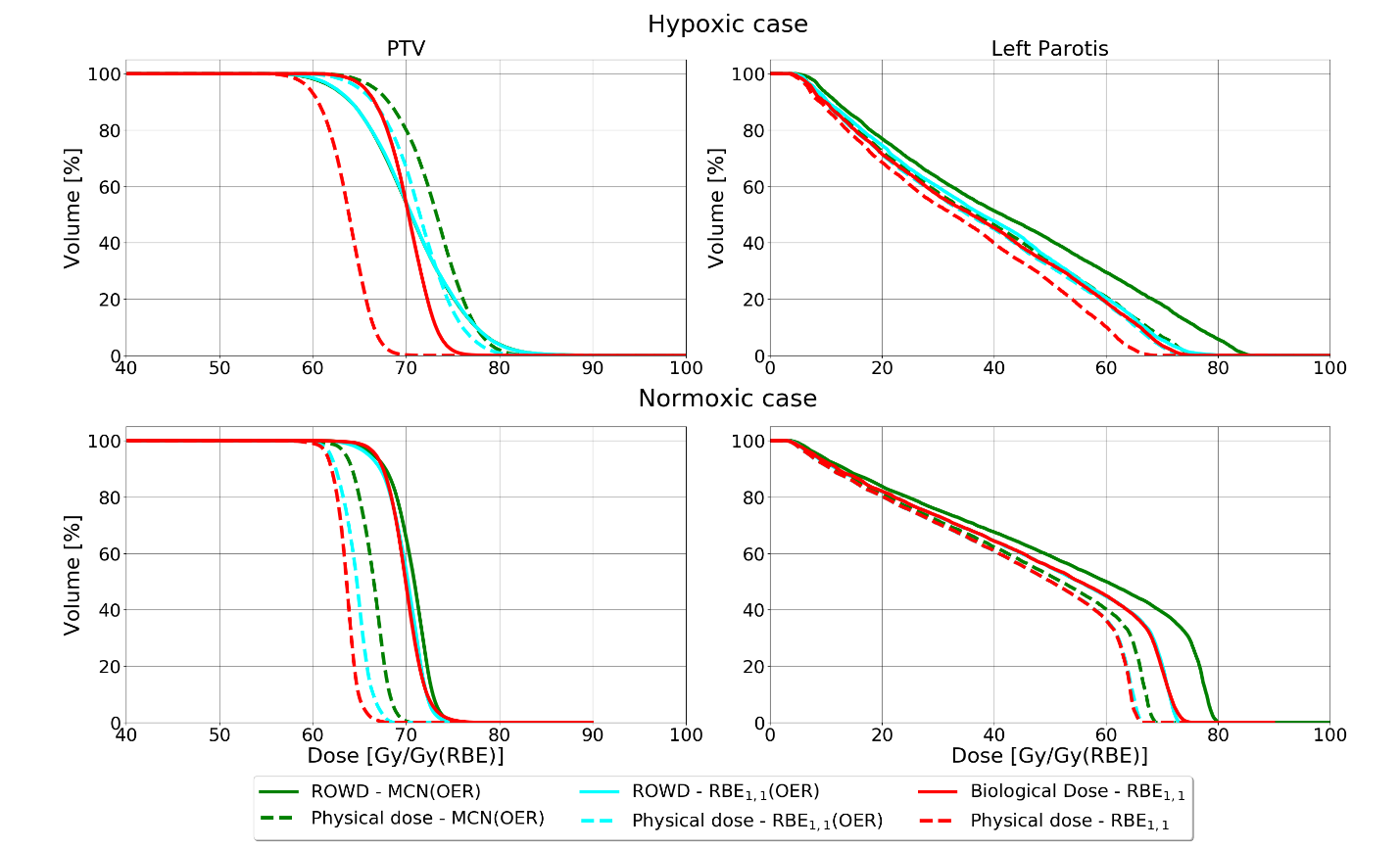


Figure A5 DVHs for the different plans for the hypoxic case (top row) and the normoxic case (bottom row) where the solid lines represent the ROWD color coded to each RBE model , while the dashed lines represent the respective physical dose using the same color coding for RBE models.


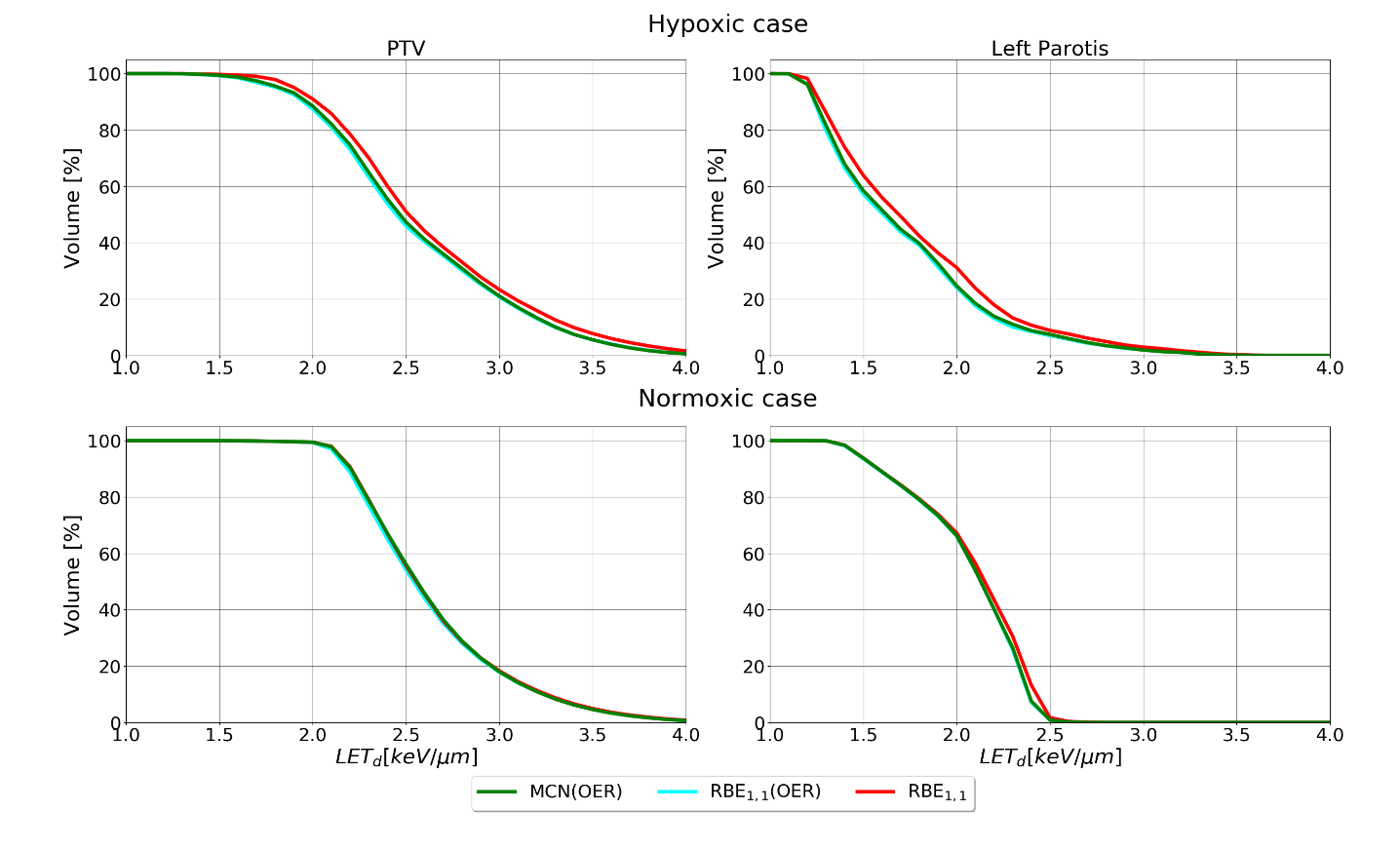


Figure A6 LET volume histogram for the hypoxic (top row) and normoxic case (bottom row).
